# Prevalence, Risk Factors, and Spectrum of Fungi in Patients with Onychomycosis in Addis Ababa, Ethiopia: A Prospective Study

**DOI:** 10.1155/2019/3652634

**Published:** 2019-06-04

**Authors:** Adane Bitew, Sinknesh Wolde

**Affiliations:** ^1^Department of Medical Laboratory Sciences, College of Health Sciences, Addis Ababa University, Addis Ababa, Ethiopia; ^2^Armauer Hansen Research Institute, Addis Ababa, Ethiopia

## Abstract

**Background:**

Onychomycosis is a common refractory infection deleteriously affecting quality of life via social stigma and upsetting day-to-day activities.

**Objective:**

To study the prevalence of onychomycosis, spectrum of fungal etiological agents, and associated risk factors.

**Methods:**

A prospective nonrandomized study on the prevalence of onychomycosis was carried out from September 2017 to April 2018 at a dermatology center in Addis Ababa. Nail scrapings were collected from 303 patients clinically identified with nail disorders of fungal origin by dermatologists. Fungal etiological agents were identified microscopically and by culture method following standard procedures.

**Results:**

The prevalence of onychomycosis was 60.4%. Fungi neither were detected nor showed visible fungal growth in 39.6% of the cases. Females were more likely to present dystrophic nails than men. Patients in the middle age group were more affected. The isolation rates of dermatophytes, yeasts, and nondermatophyte molds were 44.7%, 33.3%, and 32.3%, respectively.* Trichophyton rubrum*,* Scytalidium dimidiatum*, and* Candida albicans *were the dominant species of dermatophytes, nondermatophyte molds, and yeasts, respectively. There was no statistically significant association between onychomycosis and risk factors.

**Conclusions:**

The prevalence rate of onychomycosis in the present study was high. The isolation rate of nondermatophyte molds was comparable with that of dermatophytes. Further studies on the prevalence of onychomycosis, fungal etiological agents, and changes in species distribution of the etiological agents of nail infection in Ethiopia are important.

## 1. Introduction

Onychomycosis is a denotation used to describe fungal infection of nails in which dermatophytes, yeasts, and nondermatophyte molds have been incriminated as the etiological agents [1]. The infection affects nearly 2-9% of the general population globally [2] and the mycosis accounts for half of all nail disorders [3] and one-third of all fungal cutaneous infections [4]. Although onychomycosis is hardly life threatening, its high prevalence rate and the associated morbidity such as psychosocial effects, occupational discomfort, permanent damage to the nail, spread of the infection to other persons, and high treatment cost have made it an important public health problem [3].

The prevalence of onychomycosis particularly caused by nondermatophyte molds is increasing [5]. The aging of population, increased use of immunosuppressive drugs, an increase in the prevalence of underling disease such as HIV and diabetes that suppress the immune- status of patients, increased exposure to spas and public swimming pools, the use of tightly fitting shoes for fashions, and log-distance running in athletic games have been recognized as factor for the rise in the mycoses [6, 7].

Although the true prevalence of onychomycosis is far from resolved (i.e., prevalence figures in the literature are highly variable), the prevalence of onychomycosis, etiology, and risk factors associated with the disorder are well documented all over the world. Unfortunately, there is not a single study conducted solely on onychomycosis in Ethiopia. Socioeconomic constraints, other common prevalent health issues, and lack of expertise in the field (medical mycology) have been considered as major hindrances for such study. To this end, the main purposes of this paper were to determine the prevalence of onychomycosis, the etiology, and associated risk factors.

## 2. Materials and Methods

### 2.1. Specimen Collection and Transportation

A prospective nonrandomized study on the prevalence of onychomycosis was carried out from September 2017 to April 2018 at All African Tuberculosis and Leprosy Rehabilitation Training Center, Addis Ababa, Ethiopia, where patients with dermatological problems such as onychomycosis are referred from different health institutes in the city. A total of 303 nail scrapings were collected from clinically diagnosed patients with nail infections of fungal origin by dermatologists in duty. One clinical sample per patient was collected by scrapping from infected finger nail or toenail with sterile blade after disinfecting them with alcohol swap (isopropyl alcohol 70%, Becton Dickinson, USA). Specimens were transferred to sterile plastic petri-dishes and transported to the Department of Medical Laboratory Science, College of Health Sciences, Addis Ababa University, for further study. Clinical samples were collected after obtaining written informed consent from patients older than 16 years and assent form is completed and signed by the parents and/or guardians for those patients ≤ 16 years old. Demographic data and risk factors were collected from lab log or laboratory request form.

### 2.2. Microscopic Investigation

Clinical samples were investigated microscopically for the presence of fungal elements after grinding nail scrapings with mortar and treating them with a drop of 20% potassium hydroxide. Presence or absence of pseudohyphae or blastoconidia and shape and size of yeast were used for the detection of yeasts microscopically. Presence or absence of different types of asexual spore, the nature of reproductive structures, and shape and size of macroconidia, microconidia, and different types hyphae were used for microscopic detection of molds.

### 2.3. Culture of Clinical Specimens

A portion of each clinical sample was inoculated onto plates of mycosel agar and Sabouraud's dextrose agar containing antimicrobial antibiotics without cycloheximide (Oxoid, Basingstoke, England). All inoculated plates were kept at room temperature (25°C) for a minimum of four weeks checking frequently for any fungal growth. Molds were identified to the species and/or genus level by studying macroscopic and microscopic culture characteristics (i.e., texture, rate of growth, topography, and pigmentation) of the colonies while yeasts were identified by conventional routine diagnostic methods (i.e., assimilation and fermentation of carbohydrates and urease production). At times, urease test was used in the differentiation of* T. mentagrophytes *from* T. rubrum*.

### 2.4. Statistical Analysis

All data from the investigation were coded, double entered, and analyzed using SPSS version 20. Descriptive statistics and logistical regressions were used to estimate crude ratio with 95% confidence interval to the different variables. P-value < 0.05 was considered significant.

### 2.5. Ethics Approval and Consent to Participate

All ethical considerations and obligations were duly addressed. The study was carried out after the approval of ethical review board of the Department of Medical Laboratory Sciences (with a protocol number DRERC/309/17/MLS), School of Health Sciences, and Addis Ababa University. Data collection was started after obtaining written informed consent from study subjects and assent form was completed and signed by parents and/or guardians for those study subjects ≤ 16 years. All the information obtained from the study subjects were coded to maintain confidentially.

## 3. Results

A total of 303 clinical samples were collected from individuals with signs and symptoms of onychomycosis of which 203 (67%) were from female patients and 100 (33%) from male patients. As shown in Table 1, the highest number of patients (39.6%) was seen in the age group of 25-44 years followed by age group of 15-24 years (34.1). Onychomycosis was not significantly associated with gender (X^2^= 0.883).

Out of 303 study subjects, fungi were detected and/or isolated in 183 patients, giving a prevalence rate of 60.4%. Out of 180 culture positive samples, 163 (53.8%) yielded single colonies while 17 (21.3%) yielded mixed colonies. Among the study population, fungi neither were detected nor showed visible fungal growth in 120 (39.6%) even though samples were collected from lesions compatible to onychomycosis (Table 2).

Study subjects in the age group of 25-44 years were the most affected by onychomycosis followed by age groups of 15-24 and 45-64 years representing 120 (39.5%), 95 (31.5%), and 56(18.5%), respectively. However, bivariate statistical analysis demonstrated that age was not significantly associated with onychomycosis (Table 3). Age groups were classified following WHO age classification for health 2007 [8].

Table 4 shows the association of the various predisposing factors with onychomycosis. Among the 303 patients studied, 92 (30.4%) patients had disease other than onychomycosis. Among patients that had trauma, onychomycosis was identified in 71.9%; among study subjects with diabetes, onychomycosis was documented in 88.9%. Patients that had HIV, 71.4%, were diagnosed with onychomycosis; among those with water and soil contact, 66.7% and 85.7% had onychomycosis, respectively. Multivariate statistical analysis of these variables revealed that none of the variables were significantly associated with onychomycosis.

From 180 culture positive samples, 209 fungal species were isolated of which 87 (41.6%) were dermatophytes, 62 (33.3%) yeasts, and 60 (32.3%) nondermatophyte molds.* T. rubrum, T. mentagrophytes*, and* T. tonsurans* were the dominant dermatophytes accounting for 13.4%, 11.9%, and 9.6% of the total isolates, respectively. Among yeast isolates,* C. albicans *and* C. krusei* were predominant representing 15.9% and 9.1% of the total isolates, respectively. Of the isolates of nondermatophyte molds,* Scytalidium dimidiatum, Aspergillus fumigatus*,* and Scopulariopsis brevicaulis* were the most frequent isolates representing 5.7%, 4.8%, and 3.8% of the total isolates, respectively (Table 5).

## 4. Discussion

A precise assessment of the prevalence of onychomycosis is essential since it gives an estimate of the burden of the disease and enables estimating the potential demand for medical treatment and the economic impact of the infection. In the current study, the prevalence of onychomycosis is found out to be high (60.4%). The prevalence rates of onychomycosis in literature are highly variable. Comparatively less prevalence rates of onychomycosis, 28.3% and 56.4%% in Brazil and Tehran, have been reported by Chiacchio et al. [9] and Soltani et al. [10], respectively. Prevalence rates of 71% and 71.6% which are comparatively higher than the prevalence rate of our study have been documented in Brazil [11] and India [12]. Given that Ethiopia is a developing nation located in the tropics with wet humid climate that is conducive for fungal growth, emergence of wide spread and frequent use of communal bathing facilities in industries, sporting and leisure establishments, and low awareness of the mycosis regarding it merely as a cosmetic rather than health problem by health workers and the general population may be possible explanation for high prevalence rate of the disease in the current study.

More female study subjects were more likely to present for dystrophy than male patients. There are mixed reports about the prevalence of onychomycosis regarding gender. Brilhante et al. [13] demonstrated that females are more affected than males, while others reported that males are more prone to onychomycosis than females [14, 15]. Gender-related backgrounds have been responsible for such variations. Among these, trauma caused as the result of outdoor activities in males and hand wet work in females are the major predisposing risk factors for the development of onychomycosis. In urban Ethiopia, laundry without washing machine and house cleaning are mostly practiced by females. This may explain for a high prevalence of onychomycosis in females than males in our study.

Accurate diagnosis of onychomycosis based on signs and symptoms alone is often difficult as signs and symptoms of psoriasis of the nail, eczema, bacterial infections, and contact dermatitis mimic signs and symptoms of onychomycosis [16]. Our result supported the reports of many studies [3], because fungi neither were detected nor showed visible fungal growth in 120 (39.6%) cases even though samples were collected from lesions compatible to onychomycosis; warranting that care should be taken to correctly identify the signs and symptoms of onychomycosis from other clinical conditions that mimic onychomycosis.

Onychomycosis is reported to be more prevalent in the elderly and earlier studies have shown that there is a correlation between age and onychomycosis; see Loo [17]. The overall prevalence rate of onychomycosis in children has been reported to be 0.44% [18]. In our study, the prevalence rate of onychomycosis in patients with an age group of 1-14 was 3.9% which is comparably higher than previous study [18]. The low prevalence rate of onychomycosis in children is attributed to difference in nail plate structure, production of insufficient amounts fatty acids that have antifungal activity before adolescence, and increased growth rate of nail plate with subsequent elimination of fungus [19]. There are inconsistent reports about the prevalence of onychomycosis in adults and elders. Some authors reported higher prevalence of onychomycosis in the age group of 20-40 years [20], while others have reported a high prevalence rate above 55 years of age [21]. In the present study, out of 183 study subjects that were found out to be positive for onychomycosis, 91.9% were in the age group of 15-64 years whereas patients in the age group of 25-44 years were the most affected. However, the prevalence of onychomycosis in the age group of ≥ 65 years was about 1%. Our result was in line with that of Veer et al. [3] who reported a higher prevalence rate in patients with middle age (30-40 years), uncommon in the elderly, and least in children.

Although conflicting reports [22] are available, the prevalence of onychomycosis has been shown to be significantly higher in diabetic and HIV patients than normal individuals [23, 24], and nail trauma and environmental factors such as water and soil contact have been shown as independent risk factors for the development of onychomycosis [24]. In contrast, in the present study, we did not find any correlation with these factors, but patients with these factors were more affected by onychomycosis than those without these conditions. Differences in patient characteristics, sample size, and study design could be reasons for such discrepancy.

In the present study, in concordance with the previous reports [22, 25], it has been shown that dermatophytes were the most encountered organisms in onychomycosis followed by yeasts and then by nondermatophyte molds, respectively. However, equal incidence between dermatophytes and yeasts has been reported by Gupta et al. [26].* T. rubrum *and* T. mentagrophytes *were the most frequently isolated dermatophytes in our study.* T. rubrum *and* T. mentagrophytes *as a major cause of onychomycosis have been documented by different authors [22, 27]. Brilhante et al. [13] documented that yeasts emerged as an important cause of onychomycosis particularly in fingernails and our result was consistent with these findings. Repeated contact with water has been incriminated for an increase of yeasts in onychomycosis [22]. Interestingly, the prevalence rate of non-*albicans candida* species was almost the same as the prevalence rate of* Candida albicans *in our study. A study on vulvovaginal candidiasis and species distribution of* Candida *and their antifungal susceptibility pattern in Ethiopia [28] has also demonstrated high prevalence rates of non-*albicans candida* species,* C. krusei* being 100% resistant to fluconazole. The widespread use of fluconazole for treatment of fungal infection in Ethiopia [29] may have probably promoted selection of resistant yeasts by shifting infection to more naturally resistant species especially* C. krusei* or* C. glabrata* as suggested by Alexander and Perfect [30].

In the current study, the occurrence of onychomycosis caused by nondermatophyte molds (once considered to be contaminants) was almost equal to the occurrence of nail infections caused by dermatophytes and yeasts.* Scytalidium dimidiatum, A. fumigatus, and Scopulariopsis brevicaulis* were the commonest nondermatophyte mold species in this study representing 20%, 16.7%, and 13.3%, respectively, of the culture positive nondermatophyte molds. Comparable results to ours were found in studies conducted in Mexico, North America, and Europe [19, 31, 32]. This study also yielded fairly a large percentage of other nondermatophyte molds. Underling diseases that suppress host immunity and sustaining patients by drugs, chemicals, and mechanical processes that compromise physical barriers to infection, suppress immune mechanisms, or upset the balance of normal flora have been causing hosts to be more susceptible not only to pathogenic fungi but also to all fungi that were once considered contaminants [6, 7]. Of particular interest regarding the isolation rate of nondermatophyte molds were five- and two-time increased isolation rates of* S. dimidiatum *and* S. brevicaulis, *respectively, compared to those reported in related study conducted in Ethiopia [33]. This may indicate that the epidemiology of nondermatophyte molds appears to be changing with an increasing prevalence of* S. dimidiatum *and* S. brevicaulis.* High prevalence rate of onychomycosis and isolation of different groups of fungal species as etiological agents in the present study suggest that more studies on the prevalence of onychomycosis, the spectrum of fungal etiological agents and changes over time, and the roles of nondermatophyte fungi in onychomycosis should be conducted. This is because better understanding of these issues could lead to developing better preventive measures and, therefore, reducing the morbidly and cost of treatment incurred by the disorder.

## 5. Conclusions

The prevalence rate of onychomycosis in the present study was high. The isolation rate of nondermatophyte molds was comparable with that of dermatophytes. Further studies on the prevalence of onychomycosis, fungal etiological agents, and changes in species distribution of the etiological agents of nail infection in Ethiopia are important.

## Figures and Tables

**Table 1:** Demographic characteristics of study participants (n= 303].

Gender	Total (n, %)	Age groups*∗*				
		1-14	15-24	25-44	45-64	>65
Female	203(67)	13(4.3%)	68 (22.4%)	86(28.4)	35(11.6)	1 (0.3)
Male	100 (33)	11(3.6)	27(8.9%)	34 (11.2)	21(6.9)	7 (2.3)

Total	303	24 (7.9)	95(31.4)	120(39.6)	56(18.5)	8(2.6)

*∗*Age group- WHO age classification for health 2007 [8].

**Table 2:** Testing methods and results.

Test procedure	Numbers	Percent
KOH positive	65	21.4
Culture positive	180	59.4
KOH negative Culture positive	128	42.2
KOH positive culture negative	8	2.6
KOH and culture positive	57	18.8
KOH and culture negative	120	39.6

**Table 3:** Association of onychomycosis with age (n=303).

Age in years	Number	Onychomycosis		COR	95%CI	P-value
		Yes	No			
1-14	24 (7.9%)	12(3.9%)	12(3.9%	1		0.284
15-24	95(34.4%)	58 (19.1)	37 (12.2%)	1.317	0.537-3.231	0.547
25-44	120(39.6)	72(23.8)	48(18.5%)	1.500	0.622-3.615	0.366
45-64	56 (18.6%)	38(12.5)	18 (5.9%)	2.294	0.859-6.126	0.098
≥ 65	8 (2.6%)	3(0.99)	5(1.7%)	0.600	0.116-3.093	0.541

Total	303	183	120			

**Table 4:** Association of onychomycosis with risk factors (n=92).

Variables	Number	Onychomycosis		COR	95%CI	P-value	AOR	95%CI	P-Value
		Yes	No						
Trauma	32(34.7%)	23(71.9%)	9(28.1)	1.402	0.581-3.386	0.452		0.577-3.474	0.448
Diabetes	9(9.9%)	8(88.9)	1(11.1)	4.912	0.597-40.4447	0.139	4.955	0.600-40.929	0.137
HIV	7(7.6%)	5(71.4%)	2(28.6)	0.702	0.097-5.051	0.725	0.632	0.076-5265	0.671
Water contact	24(24.6)	19(79.2%)	5(20.8%)	1.248	0.449-3.471	0.671	1.174	0.415-3.323	0.762
Chemical contact	6(6.5%	4(66.7%)	2(33.3)	1.168	0.274-1.982	0.834	1.355	0.283-6.477	0.723
Soil contact	14(15.2%)	12(85.7%)	2(14.3%)	2.802	0.585-13.435	0.198	2.763	0.571-13.367	0.206

Total	92(30.4%	71(77.2%	21(22.8%)						

**Table 5:** Frequency and spectrum of fungal isolates in study subjects (n=303).

Fungal category	Species	Number	Percentage
Dermatophytes (87, 46.7%)	*Trichophyton rubrum*	28	13.4
	*T. mentagrophytes*	25	11.9
	*T. tonsurans*	20	9.6
	*T. soudanense*	10	4.8
	*T. schoenleinii*	2	0.96
	*T. verrucosum*	2	0.96

Nondermatophyte molds (60, 32.3%)	*Aspergillus fumigatus*	10	4.8
	*A. niger*	5	2.4
	*A. flavus*	3	1.4
	*A. terreus*	2	0.96
	Other *Aspergillus* spp.	2	0.96
	*Cladosporium spp*.	6	2.9
	*Fusarium spp.*	7	3.4
	*Alternaria spp*	5	2.4
	*Scopulariopsis brevicaulis*	8	3.8
	*Scytalidium dimidiatum*	12	5.7

Yeasts (62, 33.3%)	*Candida albicans*	33	15.9
	C*. krusei*	19	9.1
	*C. tropicalis*	4	1.9
	*Other candida spp.*	6	2.9

Total		209	

## Data Availability

The data used to support the findings of this study are included within the article.
